# LRWD1 expression is regulated through DNA methylation in human testicular embryonal carcinoma cells

**DOI:** 10.1186/s12610-021-00130-y

**Published:** 2021-05-20

**Authors:** Jui-Hsiang Hung, Han-Yi Cheng, Yung-Chieh Tsai, Hsien-An Pan, Hany A. Omar, Chien-Chih Chiu, Yin-Mei Su, Yung-Ming Lin, Yen-Ni Teng

**Affiliations:** 1 Department of Biotechnology, Chia Nan University of Pharmacy and Science, Tainan, Taiwan; 2 Department of Biological Sciences and Technology, National University of Tainan, No.33, Sec. 2, Shulin St., West Central District Tainan City, 700 Taiwan; 3 Department of Obstetrics and Gynecology, Chi-Mei Medical Center; Department of Sport Management, and Department of Biotechnology, Chia Nan University of Pharmacy and Science, Tainan, Taiwan; 4 An-An Women and Children clinic, Tainan, Taiwan; 5 Sharjah Institute for Medical Research and College of Pharmacy, University of Sharjah, Sharjah, 27272 United Arab Emirates; 6 Department of Pharmacology, Faculty of Pharmacy, Beni-Suef University, Beni-Suef, 62514 Egypt; 7 Department of Biotechnology, Kaohsiung Medical University, Kaohsiung, Taiwan; 8 Department of Obstetrics and Gynecology, Faculty of Medicine, University of British Columbia, Vancouver, British Columbia Canada

**Keywords:** LRWD1, Méthylation de l’ADN, 5-Aza-2'-déoxycytidine, Floxuridine, *LRWD1*, DNA methylation, 5-Aza-2′-deoxycytidine, Floxuridine

## Abstract

**Background:**

Sperm growth and maturation are correlated with the expression levels of Leucine-rich repeat and WD repeat-containing protein 1 (LRWD1), a widely expressed protein in the human testicles. The decrease in LRWD1 cellular level was linked to the reduction in cell growth and mitosis and the rise in cell microtubule atrophy rates. Since DNA methylation has a major regulatory role in gene expression, this study aimed at exploring the effect of the modulation of DNA methylation on LRWD1 expression levels.

**Results:**

The results revealed the presence of a CpG island up of 298 bps (− 253 ~ + 45) upon LRWD1 promoter in NT2/D1 cells. The hypermethylation of the *LRWD1* promoter was linked to a reduction in the transcription activity in NT2/D1 cells, as indicated by luciferase reporter assay. The methylation activator, floxuridine, confirmed the decrease in the LRWD1 promoter transcriptional activity. On the other hand, 5-Aza-2′-deoxycytidine (5-Aza-dc, methylation inhibitor), significantly augmented *LRWD1* promoter activity and the expression levels of mRNA and proteins. Furthermore, DNA methylation status of *LRWD1* promoter in human sperm genomic DNA samples was analyzed. The results indicated that methylation of *LRWD1* promoter was correlated to sperm activity.

**Conclusions:**

Thus, the regulation of LRWD1 expression is correlated with the methylation status of *LRWD1* promoter, which played a significant role in the modulation of spermatogenesis, sperm motility, and vitality. Based on these results, the methylation status of *LRWD1* promoter may serve as a novel molecular diagnostic marker or a therapeutic target in males’ infertility.

**Supplementary Information:**

The online version contains supplementary material available at 10.1186/s12610-021-00130-y.

## Résumé

**Contexte:**

La croissance et la maturation des spermatozoïdes sont corrélées avec les niveaux d’expression de la protéine 1 riche en répétitions Leucine et contenant des répétitions WD (LRWD1), une protéine largement exprimée dans les testicules humains. La diminution du niveau cellulaire en LRWD1 a été liée à une réduction de la croissance et des mitoses cellulaires, et à une augmentation des taux d’atrophie des microtubules cellulaires. Puisque la méthylation de l’ADN joue un rôle régulateur majeur dans l’expression des gènes, cette étude visait à explorer l’effet de la modulation de la méthylation de l’ADN sur les niveaux d’expression de LRWD1.

**Résultats:**

Les résultats ont révélé la présence d’un îlot CpP de 298 pbs (-253~+45) sur le promoteur de LRWD1dans les cellules NT2/D1. L’hyperméthylation du promoteur de LRWD1 était liée à une réduction de l’activité de transcription dans les cellules NT2/D1, comme indiqué par l’analyse de l’expression d’un gène rapporteur codant pour la luciférase. L’activateur de méthylation, la floxuridine, a confirmé la diminution de l’activité transcriptionnelle du promoteur de LRWD1. D’autre part, la 5-Aza-2'-déoxycytidine (5-Aza-dc, inhibiteur de méthylation), a significativement augmenté l’activité du promoteur de LRWD1 et les niveaux d’expression de l’ARNm et des protéines. En outre, le statut de méthylation de l’ADN du promoteur de LRWD1 dans les échantillons d’ADN génomique de sperme humain a été analysé. Les résultats ont indiqué que la méthylation du promoteur de LRWD1 était corrélée à l’activité des spermatozoïdes.

**Conclusions:**

Ainsi, la régulation de l’expression LRWD1 est corrélée avec le statut de méthylation du promoteur de LRWD1, qui a joué un rôle important dans la modulation de la spermatogenèse, de la mobilité et de la vitalité des spermatozoïdes. Sur la base de ces résultats, le statut de méthylation du promoteur de LRWD1 peut servir de nouveau marqueur diagnostic moléculaire ou de cible thérapeutique dans l’infertilité masculine.

## Background

Leucine-rich repeats and WD repeat domain-containing protein 1 (LRWD1) is a widely expressed protein in the human testicles [1, 2]. LRWD1 protein has a leucine-rich repeat (LRR) domain and three tryptophan-aspartic acid (WD40) domains. Previous studies indicated that LRWD1 serves as a scaffold for histone H3 methylation on lysine 9 in lysine methyltransferases complex [3]. Also, LRWD1 is essential for the organization of heterochromatin structure in the somatic cells [3]. In germ cells, *LRWD1* expression was observed mainly in the cytoplasm during spermatogenesis and in the neck region of mature sperm cells [4].

It was observed that the downregulation of LRWD1 expression affected cell survival and caused G1 cell cycle arrest in the human testicular embryonic carcinoma cells (NT2/D1) [4]. LRWD1 is also involved in the regulation of microtubule nucleation and cell cycle progression in NT2/D1 cells [4]. In addition, EWS-ETS fusion protein-enriched LRWD1 expression in A673/TR/shEF increased the viability of Ewing sarcoma cells [5]. Under oxidative stress, the induction of LRWD1 expression by Nrf-2 protected the cells against oxidative damage [6]. Furthermore, reactive oxygen species (ROS) and NF-κB signaling pathways were also involved in the regulation of LRWD1 expression [7]. Thus, it was hypothesized that the primary function of LRWD1 is the control of heterochromatin replication, organization, and the survival of sperm cells.

The epigenetic variations are vastly correlated with the development of many diseases such as cancer, Alzheimer’s, diabetes mellitus, cardiovascular disorders, and infertility [8–12]. In normal or cancer cells, specific gene expression regulation is mediated through epigenetic modifications, which involve DNA methylation, histone modification, and non-coding RNA [13]. Many risk factors are capable of modulating epigenetic modification, such as smoking, physical activity, nutrition, behavior, stress, and alcohol consumption [14–19]. For example, the environmental toxin, bisphenol A (BPA), decreased the number of spermatocytes and caused dysregulation of epigenetic remodeling enzymes [20]. Besides, the most representative air pollutant, benzo(a) pyrene (BaP), induced abnormal methylation of spermatozoa in rats [21]. The process of cell differentiation and embryonic development is controlled by DNA methylation, which is under the control of DNA methyltransferases and DNA demethylase. The degree of DNA methylation in sperm cells is strictly regulated, and the dynamic methylation is observed in the maturation process of sperm cells [22, 23]. During the stages of sperm cells maturation, the total CpG methylation levels are 70% in human spermatozoa yielding approximately 4% of total cytosines methylated [24]. Spermatogenesis, a dynamic DNA methylation process, is disturbed by environmental stress and epigenetic risk factors, which cause abnormal gene expression and sperm cell maturation [22, 23, 25–27].

The downregulation of LRWD1 expression was reported in the testicular tissues of patients with severe spermatogenic defects [1]. Previous studies indicated that the sperm samples of asthenozoospermia, teratozoospermia, and asthenozoospermia have significantly lower LRWD1 expression than normal subjects [1]. Decreased LRWD1 expression was also associated with structural defects in human sperm [4]. Also, the level of LRWD1 at the sperm neck was significantly reduced with a defective neck or tail in the patients with asthenozoospermia, teratozoospermia, and asthenoteratozoospermia [4]. Our previous study indicated that the expression level of LRWD1 was highly associated with cell viability in NT2/D1 cells [5]. In addition, the core region of transcription factor binding sites of the human LRWD1 gene promoter is located between − 198 to + 1 position. These observations highlighted the importance and correlation between the regulation of LRWD1 expression and male’s fertility. Since methylation participates in the regulation of gene expression, here we investigated whether methylation plays a role in the expression of LRWD1. In the current study, we have investigated the relationship between the methylation status of LRWD1 promoter and LRWD1 expression in NT2D1 cells and clinical sperm samples.

## Materials and methods

### Reagents and vectors

**F**etal bovine serum (FBS), Trypsin/EDTA solution and Penicillin-Streptomycin solution MEM media were purchased from Gibco AG (Basel, Switzerland). TRIzol reagent and lipofectamine 3000 were available from Invitrogen (Carlsbad, CA, USA). The anti-LRWD1 antibody was purchased from ABGENT (San Diego, CA USA). The anti-β-actin antibody was obtained from Santa Cruz Biotechnology (Dallas, TX USA). 30% Acrylamide/bis solution was obtained from MDBio (Taipei, Taiwan). Dimethyl sulfoxide (DMSO), Polybrene, Triton X-100, TEMED, 3-(4,5-dimethylthiazol-2-yl)-2,5-diphenyl-tertazolium bromide (MTT), floxuridine and 5-Aza-2′-deoxycytidine (5-Aza-dc) were obtained from Sigma Chemical Co. (St. Louis, MO, USA). WesternBright™ Chemiluminuescent reagent was purchased from Advansta (San Jose, CA USA). Phosphate Buffered Saline (PBS) was ordered from GeneMark (Taipei, Taiwan). Anti-mouse IgG and anti-rabbit antibodies were from Jackson ImmunoResearch (West Grove, PA, USA). *Pfu* Ultra II Fusion HS DNA polymerase was from Agilent Technologies (Santa Clara, CA USA). Gene-Spin™ 1–4-3 DNA Purification Kit-V2 was from Protech Technology (Taipei, Taiwan). CpG methyltransferase and S-Adenosylmethionine were purchased from New England Biolabs (Ipswich, MA USA). Dual-Glo™ Luciferase Assay System was ordered from Promega (Madison, WI, USA). The pCpGL-basic plasmids (provided by Dr. Michael Rehli) were reporter plasmids completely devoid of CpG dinucleotides and contained a multiple cloning site (MCS) upstream of the secreted luciferase reporter gene. So pCpGL-basic plasmids were used to study the effect of CpG methylation on *LRWD1* promoter.

### Cell incubation

The human NT2/D1 (BCRC number: 60356) cells were available from the Bioresource Collection and Research Center (Hsinchu, Taiwan). The cells were maintained in Dulbecco’s Modified Eagle′s Medium (DMEM) supplemented with 10% FBS, 100 unit/ml penicillin, and 100 μg/ml streptomycin. NT2/D1 cells were cultured at 37 °C in a humidified incubator of 5% CO_2_. The cell culture medium was replaced with a fresh medium every 2 days.

### Promoter construct

To investigate the relationship between LRWD1 expression and promoter methylation, about 500 base pairs (− 400 ~ + 93) of LRWD1 promoter from the National Center for Biotechnology Information (NCBI) were analyzed by EMBOSS Cpgplot software to identify and plot CpG islands in nucleotide sequence(s)(https://www.ebi.ac.uk/Tools/seqstats/emboss_cpgplot/).

The construct, pCpGL-h*LRWD1*, containing the LRWD1 promoter and part of the gene body (spanning from − 400 ~ + 93) was generated by PCR amplification using PrimeSTAR GXL DNA polymerase (Takara Bio Inc., Otsu, Japan). The primer sequences for cloning were: forward, 5′- CG-GGATCCCCCCACTCCCAAGCCCG − 3′ and reverse, 5′- CCC-AAGCTTTGTGGCGTCGCCCTGCG − 3′ for pCpGL-h*LRWD1*. DNA insertion was confirmed by DNA sequencing and subcloned into pCpGL-basic luciferase reporter vector (gift from M. Rehli’s laboratory, Regensburg, Germany) with *BamH*I /*Hind*III restriction enzyme cutting sites, respectively.

### Promoter analysis

The sequence of the *LRWD1* promoter region, prospective transcriptional factor binding sites for NF-κB and Nrf2, was predicted by the PROMO 3.0 Prediction Server (http://alggen.lsi.upc.es/cgi-bin/promo_v3/promo/promoinit.cgi?dirDB=TF_8.3). The pRL-TK Renilla luciferase vector (Promega Corp., Madison, WI, USA) was served as a control. Co-transfection of 2 μg pCpGL-basic or pCpGL-h*LRWD1* promoter − 400/+ 93 (pCpGL-h*LRWD1*) plasmids with 0.2 μg pRL-TK vector in NT2/D1 cells was by using lipofectamine 3000 in 6-well plates. The total cell lysate was collected after 24 h incubation, and luciferase activity was determined by the dual-luciferase reporter assay kit.

### Promoter methylation assay

In order to investigate the effect of methylation status on LRWD1 promoter activity, 5 μg pCpGL-h*LRWD1* plasmid DNA was incubated with a reaction solution containing 1 μL CpG methyltransferase (4 U/μL, New England Biolabs), 5 μL NEB Buffer 2(10x), 0.25 μL 32 mM S-Adenosylmethionine (SAM; New England Biolabs) in in a volume of 50 μL. The reaction mixture was incubated at 37 °C for 2 h and then added 0.25 μL 32 mM SAM in the reaction mixture for 2 h at 37 °C. After the reaction, the pCpGL-h*LRWD1* plasmids were purified by Gene-Spin™ 1–4-3 DNA Purification Kit (Protech Technology Enterprise, Taipei, Taiwan). In order to check the methylation status on pCpGL-h*LRWD1* plasmid, the plasmids were treated with *BstU*I restriction enzyme for 2 h at 60 °C, and DNA product was analyzed by 1.2% agarose gel electrophoresis (Supp. data A). In addition, the methylation status of pCpGL-h*LRWD1* DNA-transfected NT2D1 cells, which were treated by 5 μM floxuridine (methylation activator) or 5 μM 5-Aza-dc (methylation inhibitor), was rechecked by methylation-specific PCR (MS-PCR) after bisulfite modification. The primer sequences for MS-PCR were: forward, 5′-GGTTTCGTTTTTTTTCGGTC-3′ and reverse, 5′- CGCCCTACGTCTCCTAAAAC-3′ and then was analyzed by 3.0% agarose gel electrophoresis (Supp. data B).

### Western immunoblot analysis

Total lysates of NT2/D1 cells was extracted by using ice-cold cell lysis buffer (50 mM TRIS-hydrogen chloride, pH 7.4, containing 150 mM sodium chloride, 1 mM EDTA, 1 mM EGTA, 1.2% Triton X-100, 0.5% sodium deoxycholate, 0.1% SDS,and 1 mM PMSF) (Enzo Biochem, Inc., Farmingdale, NY, USA). Fifty microgram lysate was analyzed by SDS-polyacrylamide gels and electrophoretically transferred to a PVDF membrane (Bio-Rad Laboratories, Hercules, CA, USA). The PVDF membrane was incubated with TBST (Tris 50 μM, NaCl 0.15 M, Tween 0.1% (v/v)) containing 5% skimmed milk for 1 h at room temperature. The membranes were incubated with β-actin and LRWD1 antibodies in TBST containing 1% skimmed milk at 4 °C overnight. The PVDF membrane was washed with TBST buffer three times for 30 min. The secondary antibodies of anti-mouse IgG or anti-rabbit IgG (1:2000 dilutions) in TBST buffer were added to PVDF membrane at room temperature for 1 h. The blots were visualized with ECL Western blot detection system according to the manufacturer’s instructions (GE, Pittsburgh, PA, USA).

### Human subjects and semen sample collection

All clinical semen specimens came from Taiwan Chi mei Medical Center and were approved by the IRB. (IRB approval number: Taiwan, Taiwan Chi mei Medical Center, IRB09808–006). The experimental procedure is based on the scheme recommended by using the modified Neubauer chamber WHO criteria of the World Health Organization [28]. Semen samples were collected from 80 volunteers aged about 25–45 years old, and these volunteers required sexual restraint within 2 to 7 days. Semen analysis was performed by employing a computer-assisted semen analysis (CASA) system (Cell Motion Analyzer, SM-CMA) compared to visual estimation by microscope. All experiments were analyzed by two separate centrifugal semen samples (3000 μg, 15 min).

### Bisulfite modification and DNA methylation detection by real-time methylation-specific PCR

To detect the methylation status on *LRWD1* promoter, bisulfite modification and real-time methylation-specific PCR (MS-QPCR) were used for the evaluation of DNA methylation status on *LRWD1* promoter. The bisulfite modification was performed using EZ DNA Methylation™ Kit (Zymo Research, Orange, CA, USA). Briefly, 200–500 ng genomic DNA from sperm was mixed with 5 μl M-Dilution Buffer and adjusted to a total volume of 50 μl with sterile water. The mixture was incubated at 37 °C for 15 min and then added 100 μl CT Conversion Reagent in the mixture. The mixture was placed in a dark place at 50 °C for 12 to 16 h, and then the solution was incubated at 0 ~ 4 °C for 10 min. The mixture was added with 400 μl M-Binding Buffer and DNA samples were collected with Zymo-Spin™ IC Column according to the protocol of EZ DNA Methylation™ Kit. In the DNA methylation status assay, the primers of MS-QPCR were designed by using Methyl Primer Express Software (Applied Biosystems). In addition, hypermethylation of MyoD1 promoter was observed in many tissues and tumor cells. Therefore, MyoD1 was served as an internal control in DNA methylation assay. The PCR primers used were as follows: *LRWD1* methylation-specific primer (F) 5′- GGTTTGGGTTTCGTTTTTTTTC -3′/(R) 5′-TCGCCCTACGTCTCCTAAAAC -3′; *LRWD1* unmethylation-specific primer (F) 5′- GAGGTTTGGGTTTTGTTTTTTTTT -3′/(R) 5′-TCACCCTACATCTCCTAAAACC − 3; *MyoD1* primer (F) 5′- CCAACTCCAAATCCCCTCTCTAT -3′/(R) 5′- TGATTAATTTAGATTGGGTTTAGAGAAGGA − 3′.

### Statistical analysis

Data were presented as mean ± standard deviations from three or four independent experiments. Results were analyzed by ANOVA followed by Dunnett’s post-hoc test, and differences were considered significant at *P < 0.05, **P < 0.01 and ***P < 0.001, respectively. The methylation status of LRWD1 promoter by MS-QPCR results are expressed as △Ct ratios between the *LRWD1*and *MyoD1* control values. Pearson correlation analysis evaluated the association of the methylation status of LRWD1 promoter with (A) c-motile (%) and (B) c-static (%) for sperm motility data from CASA. The statistical analyses were performed by GraphPad Prism 5.0.

## Results

### Prediction of methylation sites on LRWD1 promoter

**Fig. 1:**
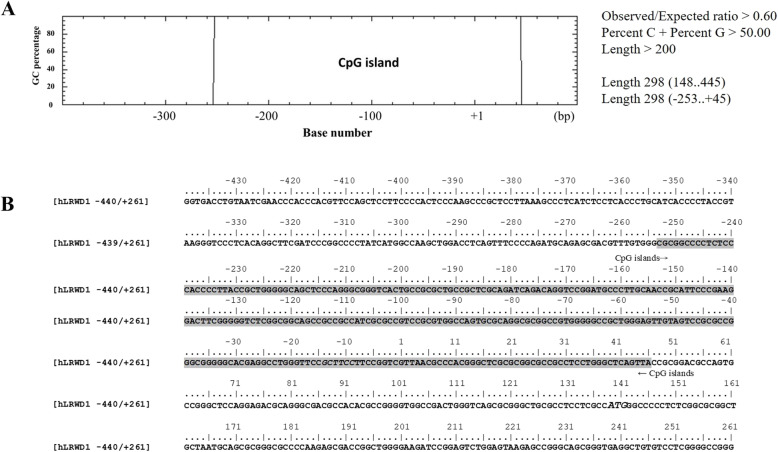
CpG island for LRWD1 promoter. **a** The CpG island of LRWD1 was predicted by online EMBOSS Cpgplot bioinformatics tools (http://www.ebi.ac.uk/emboss/cpgplot/). CpG islands of unusual CG composition Sequence from − 253 to + 45 bp (298 bps, Criteria used: Island size > 200, GC Percent > 50.0, Obs/Exp > 0.60). The transcription initiation site (TSS) is assigned nucleotide + 1. **b** The sequence of *LRWD1* promoter region was predicted by the PROMO 3.0 Prediction Server (http://alggen.lsi.upc.es/cgi-bin/promo_v3/promo/promoinit.cgi?dirDB=TF_8.3). The CpG islands are indicated by arrows and shaded. LRWD1, Leucine-rich repeat and WD repeat-containing protein 1. CG, cytosine guanine

Thus, the methylation sites on LRWD1 promoter were analyzed by EMBOSS Cpgplot software to identify and plot CpG islands in nucleotide sequence(s) (https://www.ebi.ac.uk/Tools/seqstats/emboss_cpgplot/). The transcription initiation site (TSS) was assigned nucleotide + 1. As shown in Fig. 1a, the analysis showed that there is a high proportion of CpG in the region of LRWD1 promoter with about 500 base pairs (− 400 ~ + 93). The CpG islands of unusual CG composition Sequence from − 253 to + 45 bp (298 bps, Criteria used: Island size > 200, GC Percent > 50.0, Obs/Exp > 0.60). Besides, there are many transcription factor binding sites presented in this region containing a high frequency of CpG dinucleotide (Fig. 1b). It was speculated that the LRWD1 gene is likely to be regulated by methylation.

### The effect of methylation on LRWD1 promoter activity

**Fig. 2:**
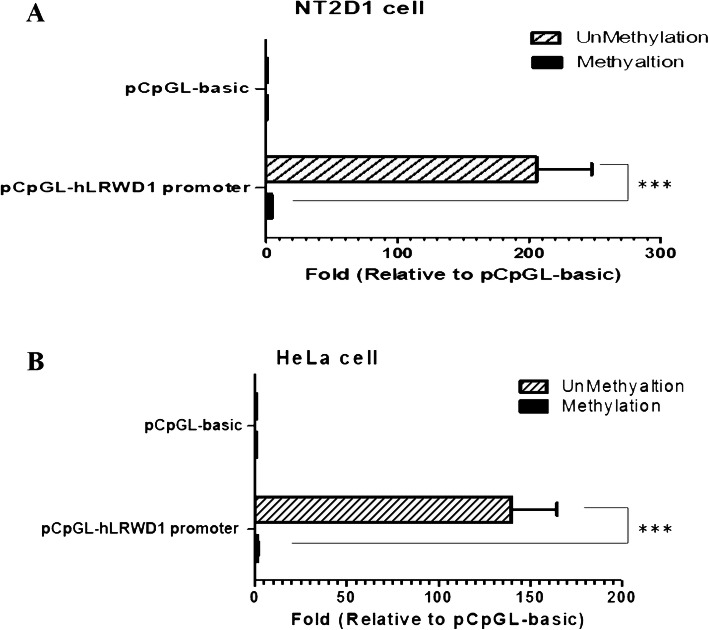
The reduction of LRWD1 promoter activity by DNA methylation in NT2/D1 cells. Two pCpGL-basic and pCpGL-h*LRWD1* plasmids were treated with or without methyltransferase. After DNA methylation assay, the methylated or unmethylated plasmids were transfected into (**a**) NT2/D1 and (**b**) Hela cells. The promoter activity was assessed by luciferase reporter gene assay, and luciferase activities were normalized to pRL-TK reporter activities and presented as fold induction compared with the empty vector (control). Columns, mean of three independent experiments; bars, SD (***, *p* < 0.001, one-way ANOVA). pRL-TK, the thymidine kinase promoter-Renilla luciferase reporter plasmid. LRWD1, Leucine-rich repeat and WD repeat-containing protein 1. SD, Standard deviation

To investigate the effect of methylation on *LRWD1* promoter activity, promoter methylation assay was employed using CpG methyltransferase. The pCpGL-basic vector is a vector with a luciferase reporter gene. This vector’s sequence backbone has no CpG to avoid the methylation of CpG in the reporter gene methylation treatment. The pCpGL-hLRWD1 plasmids were examined with (Supp. data A, lane 1, 2) or without (Supp. data A, lane 3) the methylation treatment. Then the methylated pCpGL-hLRWD1 plasmids (Supp. data A, lane 2) and methylation-specific PCR (MS-PCR) (Supp. data B) were applied in LRWD1 promoter activity analysis by luciferase reporter assay. Results indicated that *LRWD1* promoter activity decreased significantly by methylation of CpG on *LRWD1* promoter in NT2/D1 cells (Fig. 2a). In addition, the same results were observed in Hela cells (Fig. 2b). These results indicated that the methylation status of *LRWD1* promoter plays a role in LRWD1 activity.

### DNA methylation activators or inhibitors modulate LRWD1 promoter activity

**Fig. 3:**
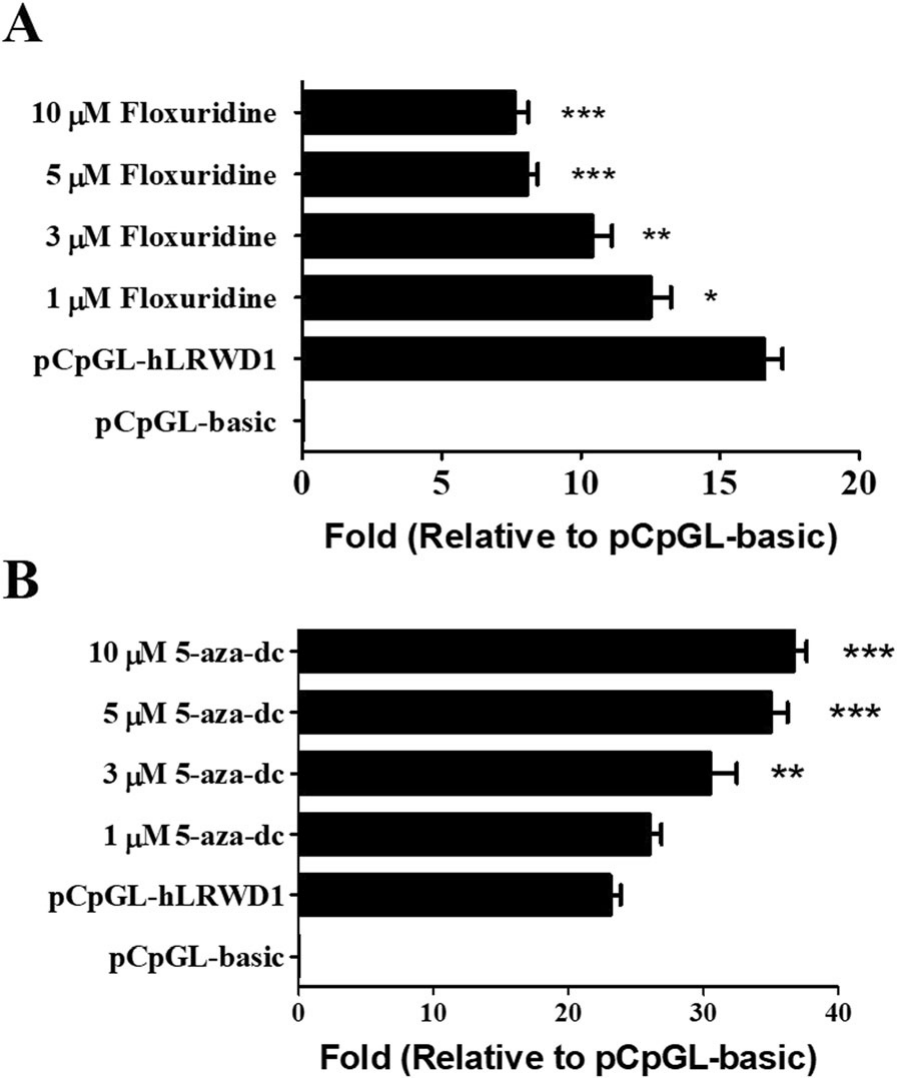
Regulation of LRWD1 promoter activity and LRWD1 expression by methylation activators or inhibitors. NT2/D1 cells were co-transfected with pCpGL-h*LRWD1* and pRL-TK reporter plasmids, and the cells were incubated with (**a**) a methylation activator (floxuridine) or (**b**) a methylation inhibitor (5-Aza-2′-deoxycytidine; 5-Aza-dc) for 24 h at the indicated doses. The promoter activity was assessed by luciferase reporter gene assay, and luciferase activities were normalized to pRL-TK reporter activities and presented as fold induction compared to the empty control vector. Columns, mean of three independent experiments; bars, SD (**, *p* < 0.01; ***, *p* < 0.001, one-way ANOVA). Columns, mean of three independent experiments; bars, SD (*, *p* < 0.05; ***, *p* < 0.001, one-way ANOVA). pRL-TK, the thymidine kinase promoter-Renilla luciferase reporter plasmid. LRWD1, Leucine-rich repeat and WD repeat-containing protein 1. SD, Standard deviation

**Fig. 4:**
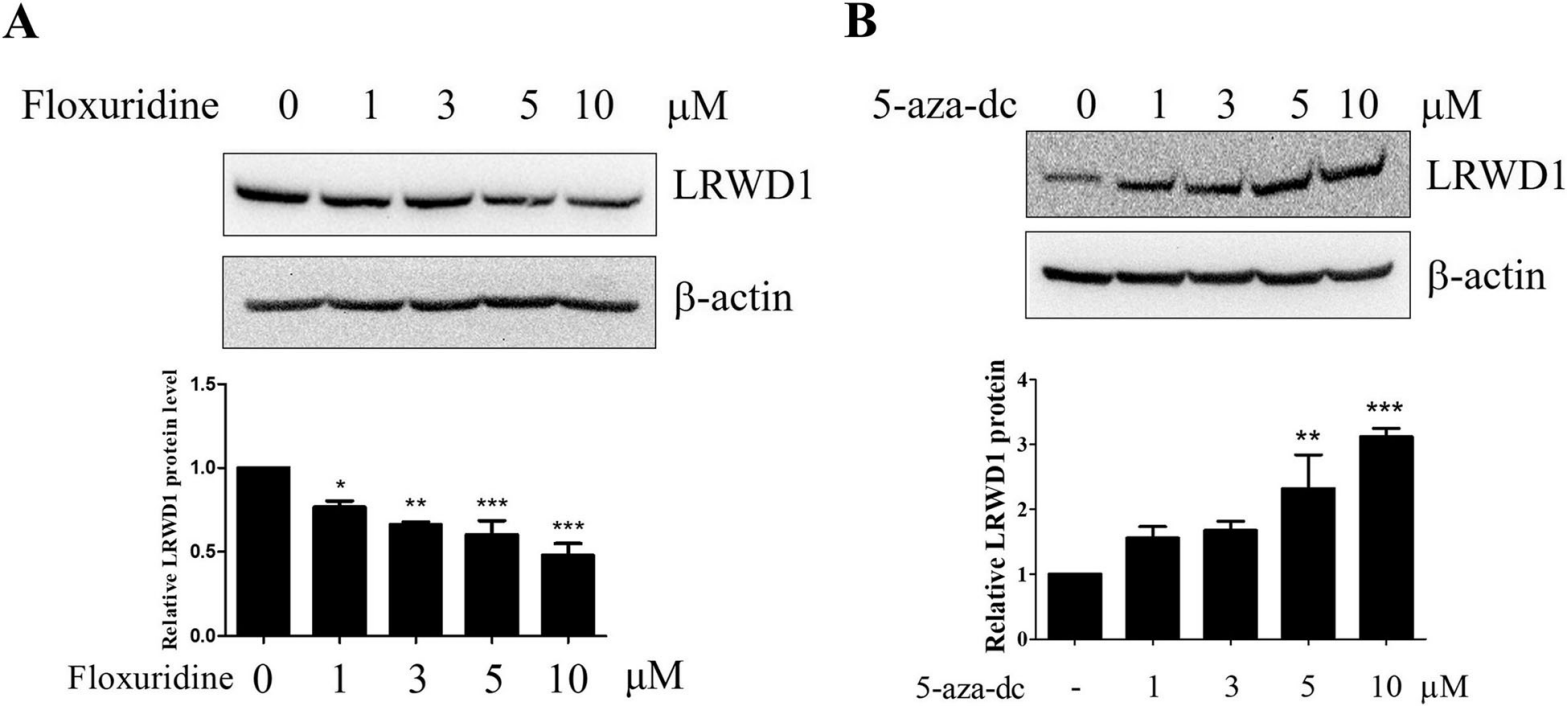
The regulation of LRWD1 expression by methylation activators and inhibitors in NT2/D1 cells. To detect the effect of (**a**) a methylation activator, floxuridine and (**b**) a methylation inhibitor 5-Aza-dc on LRWD1 expression, NT2/D1 cells were treated with 1, 3, 5 and 10 μM floxuridine or 5-Aza-dc for 24 h. Total lysates were evaluated by Western blotting. The expression levels of LRWD1 were quantified using ImageJ software. The data represent the mean of LRWD1 protein expression level from three independent experiments. Columns, mean; bars, SD (*n* = 3). Significant differences (*, *P* < 0.05; **, *P* < 0.01 and ***, *P* < 0.001, one-way ANOVA) between the control and experimental group are marked with asterisks. 5-Aza-dc, 5-Aza-2′-deoxycytidine. LRWD1, Leucine-rich repeat and WD repeat-containing protein 1. SD, Standard deviation

To confirm the role of methylation on the activity of *LRWD1*, the DNA methylation activator (floxuridine) [29] and inhibitor (5-Aza-2′-deoxycytidine, 5-Aza-dc) [30–32] were used for *LRWD1* promoter activity analysis. For DNA methylation assay, NT2/D1 cells were transfected with pCpGL-basic or pCpGL-h*LRWD1* promoter plasmid, and transfected cells were treated with or without floxuridine, the DNA methylation activator, at different doses [29]. The *LRWD1* promoter activity was analyzed by luciferase reporter assay. The results indicated that floxuridine decreased *LRWD1* promoter activity in a dose-dependent manner (Fig. 3a). On the other hand, the incubation with different concentrations of 5-Aza-dc, the DNA methylation inhibitor, significantly enhanced *LRWD1* promoter activity (Fig. 3b). In addition, the methylation status of pCpGL-h*LRWD1* DNA in transfected NT2D1 cells, which were treated by 5 μM floxuridine (methylation activator) (Supp. data B, lanes 1) or 5 μM 5-Aza-dc (methylation inhibitor) (Supp. data B, lanes 2), was rechecked by methylation-specific PCR (MS-PCR) after bisulfite modification and then was analyzed by 3.0% agarose gel electrophoresis (Supp. data B). Furthermore, Western blot analysis revealed the ability of floxuridine and 5-Aza-dc to significantly decrease and increase LRWD1 protein expression in NT2/D1 cells, respectively (Fig. 4a, b). Therefore, DNA methylation modulators can regulate LRWD1 expression.

### Sperm mobility is correlated with DNA methylation status of LRWD1 gene

**Fig. 5:**
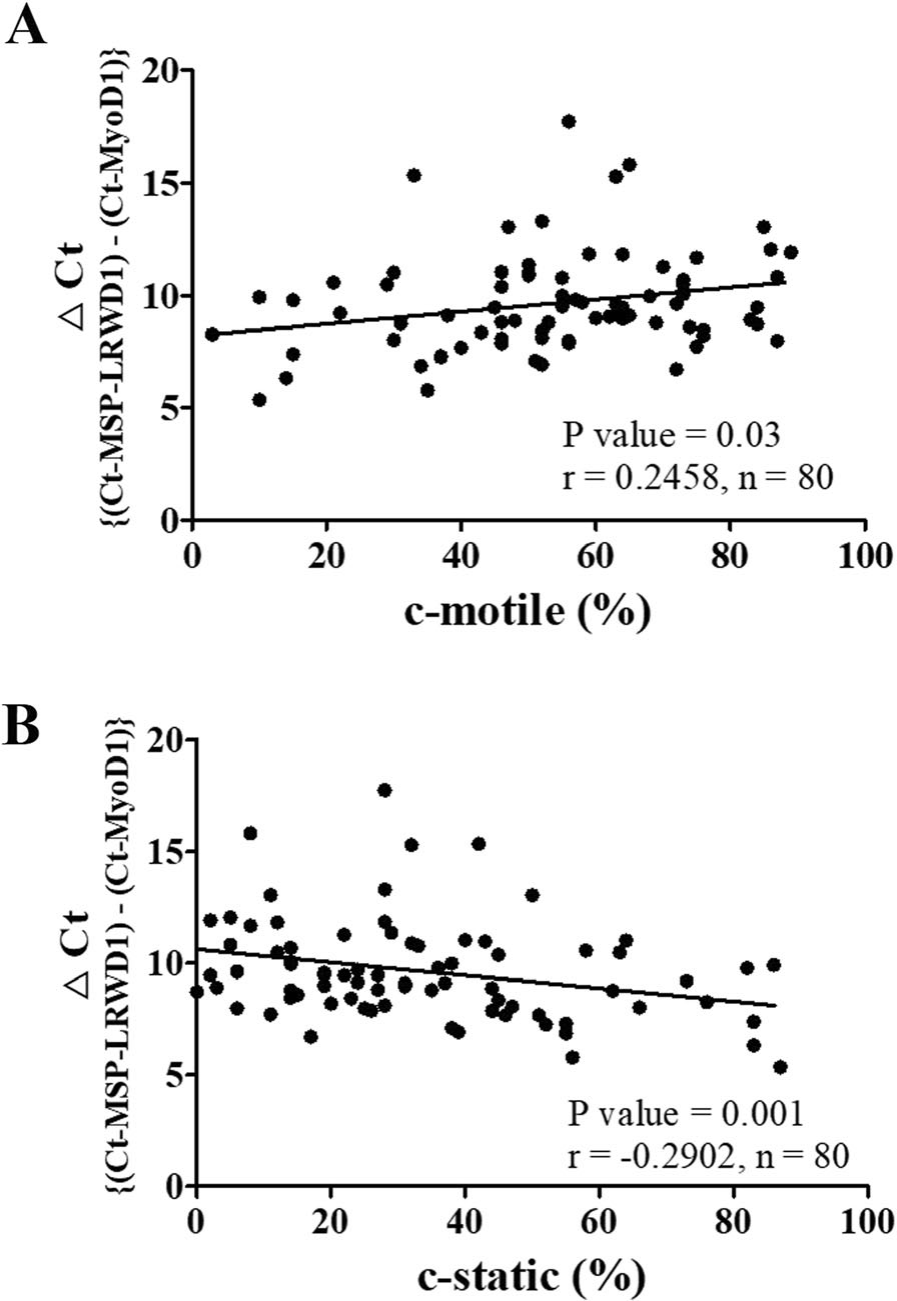
The methylation status of *LRWD1* promoter is correlated with sperm motility. The clinical samples of sperm genomic DNA were subjected to bisulfite modification and real-time methylation-specific PCR for the detection of DNA methylation status of *LRWD1* promoter. The results are expressed relative expression as ratios between the *LRWD1* values and the *MyoD1* control values. Pearson correlation analysis evaluated the association of the methylation status of *LRWD1* with (**a**) c-motile (%) and (**b**) c-static (%) for sperm motility data from semen analysis by computer-assisted semen analysis (CASA) system. r: Correlation Coefficient; *P* < 0.05: Significant differences (*n* = 80). MS-QPCR, real-time methylation-specific PCR. LRWD1, Leucine-rich repeat and WD repeat-containing protein 1. MyoD1, Myoblast determination protein 1

The DNA methylation status of *LRWD1* promoter in human sperm genomic DNA samples was examined by DNA bisulfite conversion. Bisulfite modification and MS-QPCR were used for the detection of DNA methylation status of *LRWD1* promoter. *MyoD1* gene is highly methylated in sperm, and so it served as an internal control in DNA methylation detection assay. A high Ct value indicates low methylation status of *LRWD1* promoter; on the contrary, a low Ct value indicates high methylation. The methylation status of *LRWD1* promoter by MS-QPCR results are expressed as △Ct ratios between the *LRWD1* values and the *MYOD1* control values. A high △Ct value was correlated with high sperm motility (low methylation status and high c-motile) (r = 0.2458, *p* = 0.03) (Fig. 5a). On the contrary, a low △Ct value was correlated with low sperm motility (high methylation status and high c-static) (r = − 0.2902, *p* = 0.001) (Fig. 5b). The results indicated that methylation of *LRWD1* promoter was correlated to sperm activity.

## Discussion

Our previous study has indicated that the expression levels of *LRWD1* gene is correlated in the process of sperm growth and maturation [2]. In addition, the analysis of *LRWD1* expression in the testicular tissues of normal and hypospermatogenesis patients using cDNA microarray indicated that LRWD1 mRNA expression is significantly reduced in hypospermatogenesis patients [1]. Furthermore, low expression levels of LRWD1 were observed in asthenozoospermia, teratozoospermia, asthenoteratozoospermia patients [1]. In addition, LRWD1 expression was in the centrosome [2]. Our previous study indicated that both Nrf2 and NF-κB signaling pathways have participated in the regulation of LRWD1 expression in NT2/D1 cells [6, 7]. In this study, we demonstrated that DNA methylation plays a vital role in the management of *LRWD1* expression (Figs. 2, 3). We used the pCpGL-h*LRWD1*, which would not be affected by methylation, in promoter activity assay with methylation activator/inhibitor to prove that the promoter activity of LRWD1 is affected by methylation. This result is consistent with LRWD1 protein expression in vivo by Western blot assay. The correlation between *LRWD1* expression and sperm motility by MS-QPCR showed that sperm motility was negatively correlated with *LRWD1* promoter methylation, that is, when semen *LRWD1* promoter methylation increases ((low △Ct), low sperm motility (Fig. 5a), and high stationary sperms (Fig. 5b), which means that the expression of *LRWD1* also affected the sperm motility.

LRWD1 promoter (− 198 ~ + 1) is located in the CpG islands (− 253 ~ + 45). Hypermethylation of CpG dinucleotides within gene promoters is known to be associated with gene expression repression [33]. DNA methylation is catalyzed by DNA methyltransferases on CpG nucleotides [33]. This process plays a crucial role in the regulation of gene expression during carcinogenesis and spermatogenesis [34, 35]. In addition, germ cells exhibited different DNA methylation patterns compared to other cells during the development of mouse embryo [36]. Three major spermatogenesis-associated genes, Apo A1, Oct3/4, and Pgk-2 were reported to regulate male germ cell development and differentiation in the mouse through methylation [37]. In this study, we demonstrated that the methylation status of *LRWD1* gene is associated with sperm motility and viability (Fig. 5).

The epigenetic changes play a vital role during spermatogenesis; we hypothesized that methylation-regulated *LRWD1* expression is one of the important factors for spermatogenesis. The methylation level is affected by environmental factors, so *LRWD1* can be used as one of the epigenetic and environmental markers for methylation in germ cells. It can also be utilized for the screening of some diseases, such as infertility and ROS-related disorders.

Sperm cells contain both CpG and non-CpG methylation in the DNA sequence. In the process of sperm cell maturation, the chromosomal DNA is demethylated and then methylated [38]. This dynamic methylation change, a typical process for spermatogenesis, causes global CpG methylation levels of 90% in mice and 70% in fully mature human spermatozoa [24, 39]. However, when compared to cancer cells, mature sperm cells are relatively hypomethylated [40].

In sperm cells, DNA methylation status is affected by many factors such as smoking, physical activity, and diet [14, 15]. For example, sperm cells collected after exercise training for 6 weeks or 3 months showed DNA-methylation changes in the genes involved in neurogenesis [41]. This remodeling of sperm DNA methylation enriched some non-specific gene functions such as cellular transport, localization, and metabolic processes [42]. Therefore, it is advisable to investigate whether the regulation of the methylation of *LRWD1* gene is through specific or non-specific methylation. In this study, we demonstrated that the methylation of *LRWD1* promoter region is correlated to sperm activity. Thus, the degree of methylation of the *LRWD1* gene has an impact on sperm motility (Fig. 5). Our previous study indicated that LRWD1 co-localizes with γ-tubulin in mouse spermatocyte GC-2 cells [2]. In addition, there is a high correlation between LRWD1 expression and cell cycle changes [4]. Furthermore, the WD40 domain of LRWD1 protein binds to the origin recognition complex (ORC) and plays an important role in ORC guidance to chromatin [43]. LRWD1 protein may be involved in DNA replication, repair, and cell cycle regulation [3], and so the methylation level of *LRWD1* promoter may affect the DNA replication of the cell. Since testis tissues have rapidly diving cells, the methylation of LRWD1 may be involved in the control of the DNA replication and division of these cells.

## Conclusions

This study explored the correlation between the methylation status of *LRWD1* promoter and LRWD1 expression and their impact on sperm motility. Based on the obtained results, the modulation of *LRWD1* promoter activity via methylation plays one of the vital roles in spermatogenesis. Thus, the methylation status of *LRWD1* promoter may serve as a novel molecular diagnostic or therapeutic target in male infertility.

## Supplementary Information

**Additional file 1.**

## Data Availability

The data underlying this article are available in the article and in its online supplementary material.

## Abbreviations

5-Aza-dc: 5-Aza-2′-deoxycytidine
CASA: Computer-assisted semen analysis
DMSO: Dimethyl sulfoxide
EDTA: Ethylenediaminetetraacetic acid
FBS: Fetal bovine serum
LRR: Leucine-rich repeat
LRWD1: Leucine-rich repeat and WD repeat-containing protein 1
MCS: Multiple cloning site
MS-PCR: Methylation-specific PCR
MS-QPCR: Real-time methylation-specific PCR
MTT: 3-[4, 5-dimethylthiazol-2-yl]-2, 5 diphenyl tetrazolium bromide
NCBI: National Center for Biotechnology Information
PBS: Phosphate buffered saline
pCpGL-hLRWD1: pCpGL-hLRWD1 promoter − 400/+ 93
ROS: Reactive oxygen species
WD40: Tryptophan-aspartic acid
