# Supplementary material for: LRWD1 expression is regulated through DNA methylation in human testicular embryonal carcinoma cells
Source: Basic Clin Androl. 2021 May 20;31:12. doi: 10.1186/s12610-021-00130-y (PMC8136200; doi:10.1186/s12610-021-00130-y)
Supplement: Supplementary file 1 — Additional file 1. [file 12610_2021_130_MOESM1_ESM.docx]

**Supporting Data**

**LRWD1 expression is regulated through DNA methylation in human testicular embryonal carcinoma cells**

Jui-Hsiang Hung^1,2^, Han-Yi Cheng^3^,Yung-Chieh Tsai^4^,Hsien-An Pan ^5^,Hany A. Omar^6,7^, Chien-Chih Chiu^8^**,** Yin-Mei Su^3^, Yung-Ming Lin^9^, Yen-Ni Teng^3*^

^1^Department of Biotechnology, Chia Nan University of Pharmacy and Science, Tainan, Taiwan

^2^Drug Discovery and Development Center, Chia Nan University of Pharmacy and Science, Tainan, Taiwan

^3^Department of Biological Sciences and Technology, National University of Tainan, Tainan, Taiwan

^4^Department of Obstetrics and Gynecology, Chi-Mei Medical Center; Department of Sport Management, and Department of Biotechnology, Chia Nan University of Pharmacy and Science, Tainan, Taiwan.

^5^An-An Women and Children clinic, Tainan, Taiwan

^6^Sharjah Institute for Medical Research and College of Pharmacy, University of Sharjah, Sharjah 27272, United Arab of Emirates.

^7^Department of Pharmacology, Faculty of Pharmacy, Beni-Suef University, Beni-Suef 62514, Egypt.

^8^Department of Biotechnology, Kaohsiung Medical University, Kaohsiung, Taiwan.

^9^ Department of Obstetrics and Gynecology, Faculty of Medicine, University of British Columbia, Vancouver, British Columbia Canada.

***Correspondence:** Yen-Ni Teng, Ph.D., Department of Biological Sciences and Technology, National University of Tainan, No.33, Sec. 2, Shulin St., West Central District, Tainan City 700, Taiwan. E-mail: [tengyenni@mail.nutn.edu.tw](mailto:tengyenni@mail.nutn.edu.tw)

**
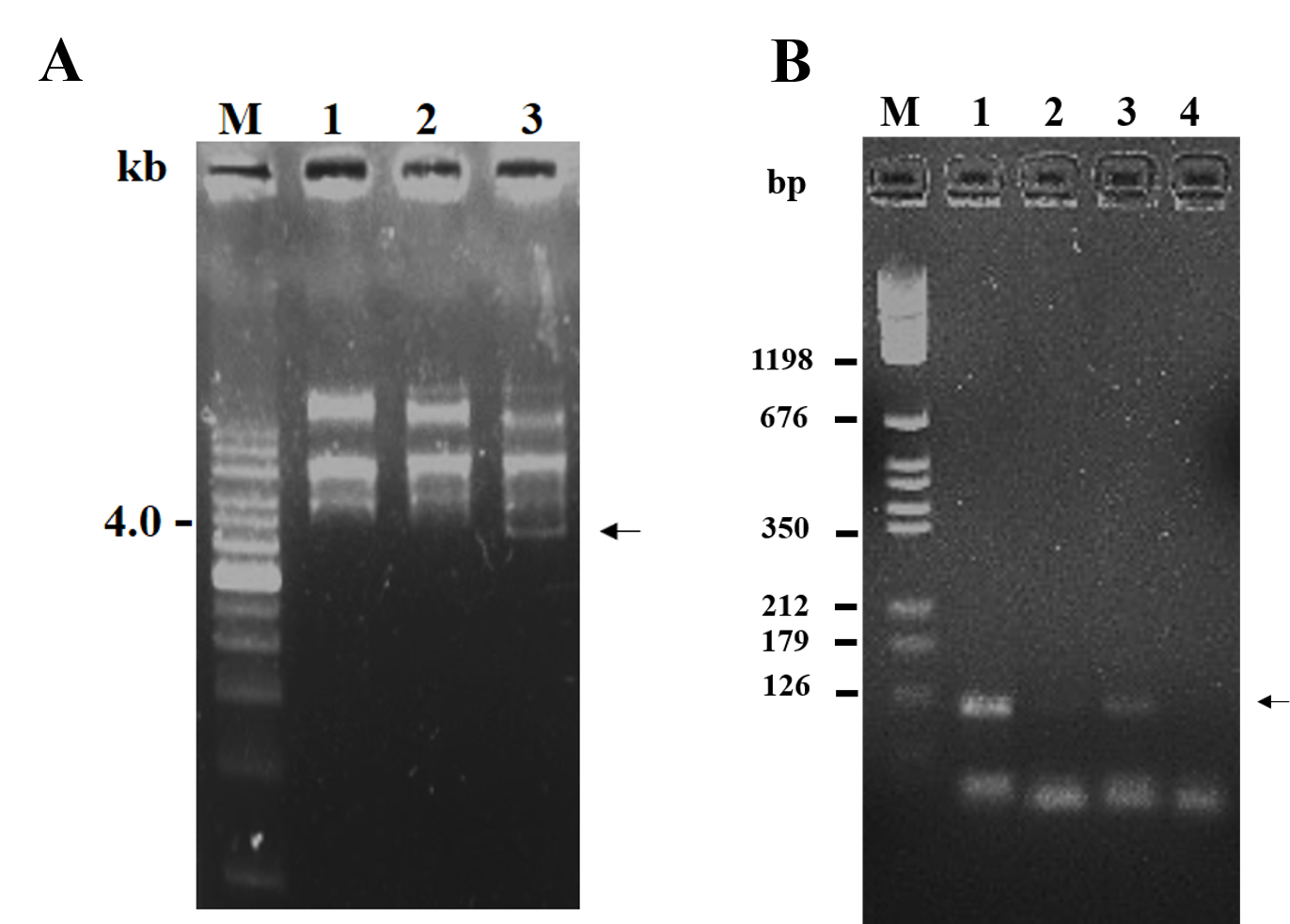
**

**Supp. Fig. 1.**

Methylation status analysis. (A) Methylation status analysis of pCpGL-hLRWD1 was incubated with (lanes 1, 2) or without (lanes 3) CpG methyltransferase and then were digested with (lanes 2, 3) or without (lanes 1) *Bst*UI restriction enzyme and fractionated on a 1.2% agarose gel electrophoresis. Lane M: size markers. Arrow indicated unmethylated DNA. (B) The methylation status of pCpGL-h*LRWD1* DNA transfected NT2D1 cells, which were treated by 5μM floxuridine (methylation activator) (lanes 1) or 5μM 5-Aza-dc (methylation inhibitor) (lanes 2) for 24 h, was rechecked by methylation-specific PCR (MS-PCR) after bisulfite modification and then was analyzed by 3.0% agarose gel electrophoresis. Arrow indicated the 112-bp product DNA of methylation-specific PCR. Lane M: size markers, Lane 3: mock control, Lane 4: PCR negative control.
